# Training in Ultrasound to Determine Gestational Age (TUDA): Evaluation of a Novel Education Package to Teach Ultrasound-Naive Midwives Basic Obstetric Ultrasound in Malawi

**DOI:** 10.3389/fgwh.2022.880615

**Published:** 2022-04-05

**Authors:** Alexandra C. Viner, Gladys Membe-Gadama, Sonia Whyte, Doris Kayambo, Martha Masamba, Enita Makwakwa, David Lissauer, Sarah J. Stock, Jane E. Norman, Rebecca M. Reynolds, Brian Magowan, Bridget Freyne, Luis Gadama

**Affiliations:** ^1^Medical Research Council (MRC) Centre for Reproductive Health, The University of Edinburgh, Edinburgh, United Kingdom; ^2^Department of Obstetrics and Gynaecology, University of Malawi College of Medicine, Blantyre, Malawi; ^3^Liverpool Clinical Trials Centre, University of Liverpool, Liverpool, United Kingdom; ^4^Mzuzu Central Hospital, Mzuzu, Malawi; ^5^Malawi Epidemiology and Intervention Research Unit, Lilongwe, Malawi; ^6^Malawi-Liverpool-Wellcome Research Programme, Blantyre, Malawi; ^7^Women's and Children's Health, Institute of Life Course and Medical Sciences, University of Liverpool, Liverpool, United Kingdom; ^8^Usher Institute, The University of Edinburgh, Edinburgh, United Kingdom; ^9^Faculty of Health Sciences, The University of Bristol, Bristol, United Kingdom; ^10^Centre for Cardiovascular Science, The University of Edinburgh, Edinburgh, United Kingdom; ^11^Borders General Hospital, National Health Service (NHS) Borders, Melrose, United Kingdom; ^12^Clinical Infection, Microbiology and Immunology, Institute of Infection, Veterinary and Ecological Sciences, University of Liverpool, Liverpool, United Kingdom

**Keywords:** gestational age, ultrasound, training, Malawi, midwives

## Abstract

**Introduction:**

Although ultrasound to determine gestational age is fundamental to the optimum management of pregnancy and is recommended for all women by the World Health Organisation, it remains unavailable to many women in low-income countries where trained practitioners are scarce. This study aimed to evaluate a novel, context-specific education package to teach midwives basic obstetric ultrasound, including the determination of gestational age by measurement of fetal femur length.

**Methods:**

The study was conducted across six sites in Malawi in January 2021. Following a virtual “training of the trainers”, local teams delivered a 10-day programme encompassing both didactic and “hands on” components. Matched pre and post course tests assessed participants' knowledge of key concepts, with Objective Structured Clinical Examinations used to evaluate practical skills. To achieve a pass, trainees were required to establish the gestational age to within ±7 days of an experienced practitioner and achieve an overall score of >65% on five consecutive occasions. A matched pre and post course survey explored participants' attitudes and confidence in performing ultrasound examinations.

**Results:**

Of the 29 midwives who participated, 28 finished the programme and met the criteria specified to pass. 22 midwives completed the matched knowledge tests, with the mean (SD) score increasing from 10.2 (3.3) to 18 (2.5) after training (*P* <0.0001). Mean difference 7.9, 95% CI 6.5–9.2. Midwives passed 87% of the Observed Structured Clinical Examinations, establishing the gestational age to within ±7 days of an experienced practitioner in 89% of assessments. Beliefs regarding the importance of antenatal ultrasound increased post course (*p* = 0.02), as did confidence in performing ultrasound examinations (*p* <0.0001).

**Conclusion:**

This study demonstrates not only that ultrasound-naive practitioners can be taught to perform basic obstetric ultrasound dating scans, confidently and competently, after 10 days of training, but also that local teams can be orientated to successfully deliver the programme virtually. Previous ultrasound training initiatives, while often more comprehensive in their syllabus, have been of considerably longer duration and this is likely to be a barrier to upscaling opportunities. We propose that this focused training increases the potential for widescale and sustainable implementation.

## Introduction

As a fundamental component of obstetric and neonatal care, the World Health Organisation (WHO) has regularly cited the need for improved estimates of gestational age in low- and middle- income countries (LMICs) as a public health priority (1–3), not only to enhance clinical care but also to strengthen the global reporting of pregnancy complications and to facilitate the evaluation of context-specific interventions to improve outcomes.

There are a number of different ways to determine gestational age but early pregnancy ultrasound is considered the most precise (4–7). Despite WHO guidance recommending that all women receive an ultrasound scan prior to 24 weeks to “estimate gestational age, improve detection of fetal anomalies and multiple pregnancies and reduce induction of labor for post term pregnancy” (8), this remains unavailable to many women living in LMICs. In these settings, gestational age is derived from either the last menstrual period (LMP) or by abdominal palpation, both of which are substantially less accurate than ultrasound (9–11). Scaled provision of ultrasound is challenging for a number of reasons (12–17) ranging from economical and geographical, to human factors and healthcare infrastructures (18). Indeed, one of the most frequently cited barriers is the lack of trained practitioners (14, 15). Despite a number of previous programmes demonstrating success in training healthcare workers to perform obstetric ultrasound, the length and complexity of many programmes has been prohibitive, with practitioners struggling to secure cover for their clinical duties in order to provide or attend training (14, 15, 19).

This study evaluated a novel training programme designed to teach ultrasound-naive healthcare practitioners' basic obstetric ultrasound, using fetal femur length to determine gestational age. We explored the ability of midwives to perform and interpret ultrasound examinations. We assessed the accuracy of their fetal measurements compared to experienced practitioners and evaluated post course changes in their knowledge and confidence. Our hypothesis was that midwives could be trained to competency within 2 weeks. We also reviewed the quality of images obtained over the following 3 months and evaluated skill retention by repeating written and practical assessments at the end of this period.

## Methods

### Pedagogical Framework

Our programme was designed to teach ultrasound-naive midwives in LMIC settings to perform the basics of obstetric ultrasound, including:

- The safe and appropriate use of an ultrasound machine
- The identification of number of fetuses
- The confirmation of fetal viability
- The confirmation of fetal presentation
- The determination of gestational age and estimated date of delivery by measurement of the fetal femur length (FL)
- The upkeep and secure storage of the ultrasound machines

The training programme was based on pilot work undertaken in Malawi in early 2020 and included both didactic and practical components. The first day and a half comprised of intense “classroom based” sessions, followed by eight and a half days of practical “hands on” experience. Details of these sessions are shown in Table 1. Simulation sessions using bespoke low-cost phantoms were incorporated into the “classroom” component, not only to help familiarize trainees with the machines, but also to help them develop confidence with probe manipulation prior to scanning volunteers. Small group sessions were intended to encourage discussion and team problem solving, as well as to encourage trainees to take ownership of the service and to anticipate and troubleshoot what they envisaged may be barriers to the implementation of ultrasound in their facilities.

**Table 1:** Components of ultrasound course.

Lectures	- Introduction to ultrasound - Scanning tips and orientation - Introduction to the ultrasound machine - How to scan for number of fetuses, fetal viability, and fetal presentation - How to scan for gestational age using fetal femur length
Small group sessions	- Incorporating ultrasound into your routine antenatal clinics - Safety and storage of the ultrasound machines
Simulation sessions	- What's in the bag? (Ice breaker) - Femur length simulators - Femur length—Good or bad?
“Hands on” practical sessions	Scan practice on client volunteers directly supervised by trainers
Formal Trainee Assessments	Observed scans formally assessed by trainers

The “hands on” sessions were conducted at the individual facilities, during which trainees had the opportunity to perform directly supervised ultrasound examinations on client volunteers. Feedback was provided in real-time and, as their skills evolved, trainees were supported to perform their scans with increasing independence. To complement the training, trainees were provided with a comprehensive printed handbook containing all of the information relayed in the lectures. Laminated sheets encompassing key concepts were also made available with each of the ultrasound machines as aide memoires.

### Competencies and Standards

**Figure 1:**
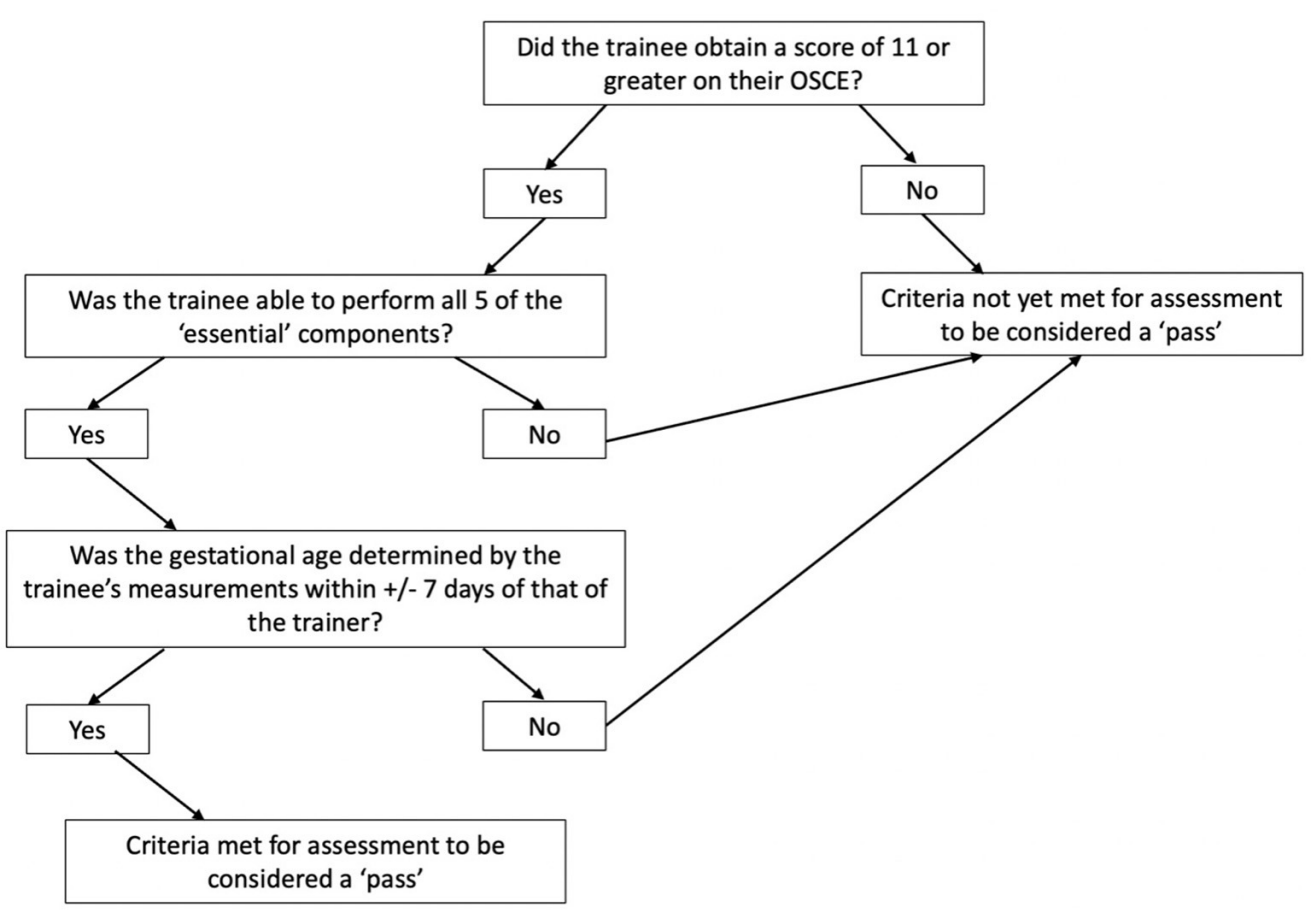
Flowchart depicting methodology of trainee assessment.

In the absence of a universally accepted definition of what constitutes competency in obstetric ultrasound, our group reached a consensus agreement based on previous initiatives (20–24). Trainees were evaluated by way of Observed Structured Clinical Examinations (OSCEs) and their ability to determine the gestational age of clients to within ±7 days of the trainers. The OSCE comprised of 17 components mapped to the curriculum, with five tasks considered “essential”. To pass, the trainee was required to achieve an overall score of 11 (65%) or greater, correctly perform all 5 of the “essential” tasks and determine gestational age to within ±7 days of the gestational age assessed by their trainer. Both trainers and trainees were blinded to the measurements and gestational age until the end of the examination. Once trainees performed five consecutive examinations fulfilling these criteria, they were deemed competent to perform these basic ultrasound scans independently. Details of the OSCE components are shown in Table 2, with the “essential” tasks depicted in bold. A flowchart illustrating the protocol for assessment of competency is shown in Figure 1.

**Table 2:** Components of OSCE.

Is the trainee able to set up and switch on the scanner?
Is the trainee able to prepare and position the client appropriately?
Does the trainee ensure that they start a new examination by pressing either “end exam” or “new patient”?
Does the trainee orientate the probe correctly?
Does the trainee assess the uterus sufficiently to establish number of fetuses?
*Does the trainee determine number of fetuses correctly?*
*Is the trainee able to identify and display the fetal heart?*
*Based on this does the trainee correctly interpret fetal viability?*
*Does the trainee correctly determine fetal presentation?*
Does the trainee optimize their images where appropriate?
Does the trainee obtain a suitable image from which to take their first measurement?
Does the trainee obtain a suitable image from which to take their second measurement?
Does the trainee obtain a suitable image from which to take their third measurement?
Is the trainee able to generate a report for their ultrasound scan?
*Does the trainee consider the LMP when interpreting the scan results and correctly determine the EDD?*
Does the trainee document their results adequately?
Does the trainee explain their results to the client?

“*Essential” tasks depicted in bold*.

### Learning Environment

#### Setting

The training was conducted in January-February 2021 as part of a “parent” study exploring what factors may influence the upscale of basic antenatal ultrasound in LMIC settings (PACTR202010788566263). The project ran across 6 health facilities in Malawi, selected to encompass both urban and rural facilities. These included 3 sites in Blantyre, 1 in Lilongwe and 2 in the northern district of Karonga.

#### Faculty

Fifteen local trainers were recruited to facilitate the course based on the following predefined criteria, kept intentionally broad to reflect the general paucity of trained practitioners in many LMIC settings. They should be an obstetrician (trainee or consultant), radiologist, sonographer, midwife or clinical officer, who has received training in basic obstetric ultrasound (via a formal or apprenticeship model) and who has performed independent obstetric ultrasound for a minimum of 1 year. They must also be capable and confident to troubleshoot any problems the trainees may encounter performing scans.

Nine of the trainers were doctors in obstetrics and gynecology, four were clinical officers, one a radiographer and one a clinical associate. Eleven had received formal training in obstetric ultrasound, with the remaining three taught “on the job”. They had an average of 5.9 years' experience in performing obstetric ultrasound, ranging from 1.5 to 16 years. All trainers attended a “training of the trainers” session, held virtually as a result of COVID-19 restrictions. All were provided with a detailed training manual and complied with the same standard operating procedures. A ratio of 1:4 trainers to trainees was maintained for all “hands on” sessions.

#### Trainees

Twenty-nine midwives were selected to participate by their local District Nursing Officer (DNO), based on their role as a key provider of antenatal care to women at the participating facilities and their engagement in service improvement. In order to preserve the continuity of subsequent scanning services, we sought to train a minimum of four midwives per site. Unfortunately, in order to ensure the ongoing provision of routine clinical care at the facilities during the training period, not all midwives at each site were able to participate. All held either a Degree or Diploma in Nursing and Midwifery and none had any previous experience of using ultrasound. They had an average of 10.5 years of experience, ranging from 1 to 30 years, representative of the skill mix working within the participating facilities.

#### Volunteers

“Hands on” sessions were made possible thanks to client volunteers, eligible to participate if they were over 18 years old, thought to be over 14 weeks gestation and able to provide informed written consent. They were provided with a small allowance to cover their travel expenses, and each was allocated a unique identification number to maintain their confidentiality.

### Follow Up and Image Review

All training was complete by the end of February 2021, with remote supervision and image review provided for the following 3 months. To assess the ongoing quality of the ultrasound examinations, trainees were required to submit images for regular review. Images were assessed by two independent reviewers who were experienced in obstetric ultrasound and blinded to the midwife submitting the image. If the reviewer was confident that the image was that of a femur, they went on to assess the quality of the image using the following criteria;

- The femur should be displayed horizontally at an angle <45 degrees
- Both ends of the femur should be clearly visible
- The femur should fill >50% of the ultrasound images
- If depicted, the calipers should be correctly placed

If the reviewer did not consider the image to be clearly that of a femur, the image was deemed unacceptable, and no further assessment of that image was performed. Of those that were rated, a score of 2 out 3 was deemed acceptable for those without calipers and 3 out of 4 if calipers were shown. Scores and comments were fed back to the trainees weekly.

### Data Collection

Prior to the training, each trainee undertook a 24-question multiple choice knowledge test to assess their theoretical understanding of ultrasound, scanning technique and basic fetal anatomy. They also completed a questionnaire using a 5-point Likert scale (1 = Strongly Disagree, 5 = Strongly Agree) to assess their attitude toward and confidence using ultrasound. Both assessments were repeated immediately after completion of the training and again 3 months later.

### Statistical Methods

Data was analyzed using SPSS (IBM SPSS Statistics for Windows, Version 24.0. Armonk, NY: IBM Corp). Matched pre and post course knowledge tests were analyzed using paired *t*-tests, with a Bland Altman plot used to assess the level of agreement between the gestational ages assigned by the trainees vs. those obtained by the trainers. Within the pre and post course questionnaires, questions evaluating similar concepts, for example confidence, were allocated into four groups for analysis, with the reliability of these groupings tested using Cronbach's alpha. If scoring >0.8, these were then analyzed as a group using a Wilcoxon signed rank test. If a group did not demonstrate adequate reliability, scoring <0.8, the statements were analyzed as single items. Intraclass correlation coefficients (ICC) were used to assess the inter-rater reliability of the reviewers assessing the follow up scans. A *p*-value <0.05 was considered statistically significant.

### Ethical Considerations

All participants (trainees and client volunteers) provided informed written consent, with participant information leaflets and consent forms available in both Chichewa and Tumbuka, as well as in English. As key stakeholders, the Ministry of Health in Malawi and the District Health Officers of the participating sites were involved in the conception of the project and supportive of its implementation. This study was approved by the University of Edinburgh and the University of Malawi—College of Medicine Research and Ethics Committee (COMREC) P08/19/2768.

## Results

At the end of the 10-day programme, 28 trainees had completed the training and all were certified as competent. The remaining trainee was unable to finish the course due to illness. Three hundred and ninety-five clients participated across the six sites, all of whom had a viable intrauterine pregnancy. No woman required referral for any suspected complication or anomaly, however 1 client was referred for high risk care following the identification of multiple pregnancy (twins). Two clients were found to be <14 weeks gestation and therefore ineligible to participate. Two hundred and twelve clients were unable to recall their LMP (54%) and 153 (39%) presented prior to 24 weeks gestation.

### Knowledge Test

Twenty-two of the 28 trainees completed matched pre and post course knowledge tests. All improved on their original score, with the mean (SD) score rising from 10.2 (3.3) to 18 (2.5) following training (*p* <0.0001). The mean difference was 7.9, 95% CI 6.5–9.2.

### Changes in Confidence and Perception

Cronbach's alpha confirmed “good” reliability within groups 1–3, as depicted in Table 3, therefore the responses to these questions were analyzed as a group. The questions allocated into group 4 however, were found to demonstrate “poor” reliability and therefore were analyzed as single statements. Twenty-three of the 28 trainees completed matched pre and post course questionnaires, the results of which are summarized in Table 3. Prior to training, most disagreed with the suggestion that ultrasound posed risks for the mother and baby, however the overall strength of this conviction increased following the programme (*p* = 0.027). Trainees' confidence in performing ultrasound examinations also increased after training (*p* <0.0001), as did their perceived ability to incorporate ultrasound into their routine work (*p* = 0.016). Their belief regarding the importance of ultrasound in antenatal care and their interest in performing scans also increased, however these results did not reach significance.

**Table 3:** Pre and post course questionnaire groupings and results.

	**Cronbach's alpha**	**Median scores (*****n*** **=** **23)**		**Total scores (*****n*** **=** **23)**		**Wilcoxon signed rank test**
		**Pre**	**Post**	**Pre**	**Post**	
**Group 1—Trainees' perceived risk of using ultrasound in pregnancy**						
1. I believe that ultrasound has risks for the mother	0.812	2	1	40	31	*p* = 0.027
2. I believe that ultrasound has risks for the baby		2	1	41	32	
**Group 2—Trainees' beliefs about the role of ultrasound in antenatal care**						
1. I believe that every pregnant woman should have an ultrasound scan	0.885	5	5	102	99	*p* = 0.092
2. I believe that providing an ultrasound scan will improve maternity care		5	5	101	110	
3. I believe that it is important to perform ultrasound scans to determine number of fetuses		5	5	99	113	
4. I believe that it is important to perform ultrasound scans to determine fetal viability		5	5	100	110	
5. I believe that it is important to perform ultrasound scans to determine gestational age		5	5	99	110	
6. I believe that midwives should perform ultrasound scans		5	5	100	111	
**Group 3—Trainee confidence in performing ultrasound examinations**						
1. I am confident in discussing the reasons for doing an ultrasound scan in pregnancy with clients	0.863	4	5	94	112	*p* = <0.0001
2. I am confident in discussing the process of doing an ultrasound scan with clients		4	5	85	111	
3. I am confident in setting up and using an ultrasound machine		3	5	73	108	
4. I am confident in cleaning and storing an ultrasound machine		3	5	73	105	
5. I am confident to perform an unsupervised obstetric ultrasound scan to determine number of fetuses		2	5	58	102	
6. I am confident to perform an unsupervised obstetric ultrasound scan to determine fetal viability		2	5	63	104	
7. I am confident to perform an unsupervised obstetric ultrasound scan to determine gestational age		2	5	59	99	
8. I am confident in asking for help		5	5	96	107	
**Group 4—Trainees attitudes toward incorporating ultrasound into their own practice**						
1. I am interested in performing basic obstetric ultrasound scans	0.554	5	5	106	112	*p* = 0.248
2. I have time to incorporate ultrasound scans into my antenatal clinics		4	5	89	106	*p* = 0.016

### Practical Skills

**Figure 2:**
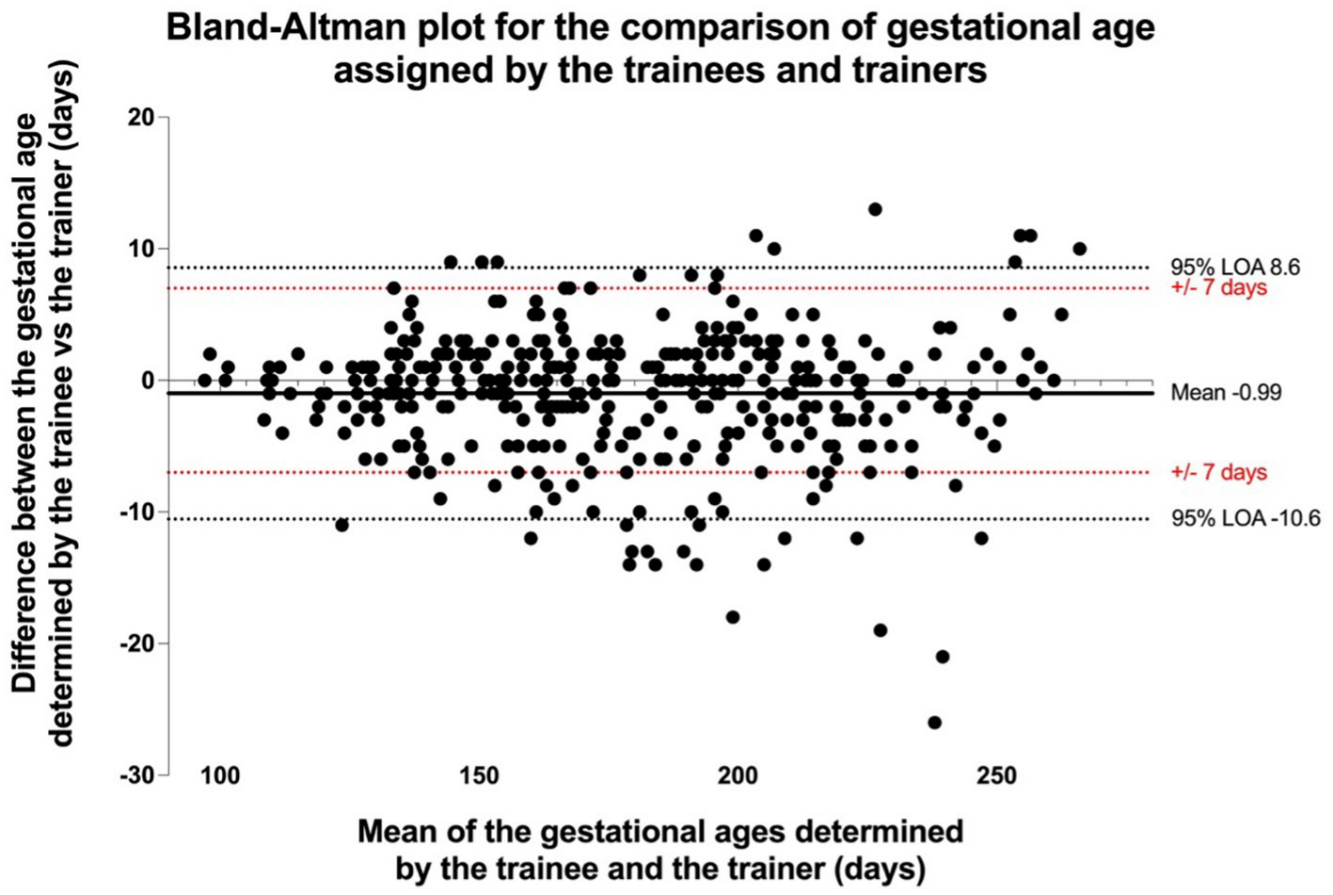
Bland Altman plot of comparison between the gestational ages assigned by the trainees vs the trainers.

Of the 405 OSCE assessments that were undertaken, 351 (86.7%) were considered a pass. Every assessment scored the required 11 or more, with 277 (56%) achieving the top mark of 17. Of the 54 assessments marked as a fail, eight were due to an inability to complete one of the “essential” tasks, 41 due to insufficient accuracy in the determination of gestational age and five on account of both. Aside from the accurate determination of gestational age, the most common problem encountered within the OSCE was the inability to correctly determine fetal presentation. Trainees were able to determine gestational age to within ±7 days in 359 (89%) of cases, with Bland Altman analysis approximating the overall level of agreement as ±9.6 days as shown in Figure 2.

### Maintenance of Skills

Due to illness and competing clinical duties, repeat assessments after 3 months were only obtained for nine trainees. Within this group, the mean score of 19 obtained at the end of the training was maintained (*p* = 0.772). Trainees maintained their beliefs regarding the importance of ultrasound within antenatal care and continued to report high levels of confidence in performing independent ultrasound examinations (*p* = 0.916 and *p* = 0.670, respectively).

Twenty-seven repeat OSCE examinations were undertaken by nine trainees. Twenty-three of the assessments met the criteria to pass with all nine trainees demonstrating retention of their practical skills. The mean (SD) OSCE score was 16.8 (0.5), with all five “essential” tasks completed successfully for every assessment. Trainees were able to determine the gestational age to within ±7 days of the trainers on 24 (86%) occasions.

### Image Review

A total of 130 images were submitted for review in the 3 months following the training. Of these, one reviewer marked 17 (13%) as unacceptable and the other 13 (10%). In 9 (6%) cases this was because the reviewers did not consider the image to be that of a femur. One reviewer awarded 67 (52%) of the images the top score, the other 57 (44%). The most common source of error was that the femur did not fill >50% of the image. Levels of agreement between the reviewers were high, as demonstrated by an ICC of 0.962 (*p* <0.0001).

### Cost

The total cost of delivering this programme across six sites was £55,182, including the provision of six Mindray DP-10 ultrasound machines.

## Discussion

We have demonstrated that it is possible to train ultrasound-naive practitioners to perform basic obstetric ultrasound scans, confidently and competently, after just 10 days of training, with skills retained after 3 months of independent practice. Despite having no prior experience in using ultrasound, all trainees displayed significant increases in their knowledge, confidence, and practical skills. These were sustained in the group available for follow up, however as this was only a third of participants this result should be interpreted with caution. Furthermore, in contrast to many other programs that have been developed in collaboration with groups from high income countries (25–31), our training was delivered exclusively by local faculty who were orientated to the programme virtually. This not only improved the immediate success of the programme, as the local team were much better placed to navigate the intricacies of the setting and troubleshoot any unexpected issues, but also makes this a more sustainable model.

This package was differentiated from previous programs described in the literature by applying a simplified approach to a similar baseline pedagogical framework (21, 23–25, 28, 31). Firstly, we chose to date pregnancies using FL as a single parameter. Compared with the conventional, more complex circumferential measurements of abdominal circumference (AC) and head circumference (HC), measurement of the FL requires only an image of a straight bone, making it a much easier measurement to obtain (6, 32). The reported difference in the gestational age assigned by FL alone is only ±2.4 days at <24 + 0 weeks, ±1.7 days at 24–29 + 6 days and ±1.8 days at >30 + 0 weeks (33). We believe that utilizing this approach has the potential to maximize the sustainability and implementation of this US training program without compromising clinical care. Secondly, in contrast to other programs (21, 30, 34–37), we purposefully excluded items such as the identification of fetal anomaly or measurement of cervical length and covered only the fundamentals of basic obstetric ultrasound. This was agreed with local stakeholders as an appropriate approach, which minimized training time and thus time away from work, while maximizing the possibility for accurate gestational age assessment in the community in line with WHO guidance.

Our program was further strengthened by the robust approach to assessment. There is a lack of consensus on the assessment of competency in obstetric ultrasound and a number of previous programs have omitted practical assessment completely (27, 31, 35, 38–40). The OSCE approach to “hands-on” assessment is well-established (24, 26, 28, 30, 41), as is the concept of comparing trainee measurements with those obtained by the faculty (20, 22, 23, 33, 42). By combining these, we sought to ensure that all aspects of our curriculum were assessed, with trainees demonstrating their ability to explain and document their findings as well as obtain accurate results. The need to achieve this on five consecutive occasions is more demanding than any previous program, but we felt that it was important to ensure trainees were able to perform consistently prior to independent practice.

Local faculty, training midwives in their own health facilities was also an important strength, not only because it supported unbroken scanning experience, but also because the faculty were familiar and trusted colleagues, from whom the midwives were comfortable seeking help. This helped to foster a supportive learning environment, exemplified by the midwives continuing to seek clarification regarding their scans when unsure, predominantly via a designated WhatsApp group (43). The local training team were also able to visit the sites intermittently to provide in person support.

Despite delivering successful training, there were some unanticipated challenges, namely power outages, transport strikes and a pandemic. Problems arising as a result of strike action were easily mitigated by adopting a pragmatic approach and moving “hands on” sessions to alternative days, although this did result in some of the post-course responses being overlooked, thus limiting our matched pairs. Although a little disruptive, this ensured adequate numbers of pregnant volunteers were available to undergo ultrasound scans. Delayed timelines as a result of COVID-19 impacted our ability to obtain follow up data for all participants and restrictions not only prevented the UK faculty from traveling to participate, but also necessitated a virtual orientation of the local team members who had not been present for the pilot training. Initially perceived as a set-back, this subsequently emerged as an important strength. Not only was virtual orientation both straightforward and effective, but the training enhanced by the exclusively local faculty, able to build on their pre-existing rapport with the midwives. Crucially, this also demonstrates that the programme can be delivered successfully by local teams out with those who developed it, a strong predictor of success in other settings.

Collating the trainees' images for remote review was also found to be an unexpected challenge. Having initially developed a bespoke mobile application to facilitate this, we found that users with non-android devices were unable to download it and were therefore unable to submit or review images. As a result, trainees were required to print a subset of their anonymized images, which were then collected in person with feedback provided a few days later. Although ultimately effective, this approach resulted in delays to feedback and entailed the ongoing provision of various consumables. Had it been possible under our ethics approval, a pragmatic approach to overcome this issue might have been to facilitate follow up via WhatsApp. Cheap and readily available, the use of WhatsApp in healthcare projects is well-established (44, 45). and this approach would be well-aligned with the concept of embedding initiatives into pre-existing systems.

Following the success of this training program, it is being evaluated as part of a mixed methods quasi-experimental trial with the primary outcome of proportion of women with accurate gestational age assessment at six health centers in Malawi. The results of this program and progress of the trial have been endorsed by the Reproductive Health Directorate of the Ministry of Health of Malawi, the Association of Malawian Midwives and the Society of Obstetricians and Gynecologists of Malawi. In addition, we have identified one trainee per site (*n* = 6) with an aptitude for scanning who will be mentored to become the next generation of trainers. Finally, in collaboration with regulatory bodies in Malawi, we are discussing a how to incorporate basic obstetric ultrasound into the nursing and midwifery curriculum in Malawi, demonstrating the ongoing local commitment to upscale this service. Although ours was an experienced group, with an average of 10.5 years clinical experience, we believe that by integrating this programme into midwifery training ultrasound skills could be made accessible to midwives at all professional levels.

## Conclusion

The TUDA training program is an effective method of training midwives in basic obstetric ultrasound in a 2 week period, thus overcoming a major barrier cited by previous initiatives (12, 14, 15, 19, 46). This program has the potential to contribute to efforts to achieve coverage of current WHO recommended guidance for basic obstetric ultrasound in LMICS. By empowering developing local faculty to provide supportive supervision in the trainees primary place of work, local acceptability was increased and basic implementation hurdles overcome during the training phase facilitating progression to independence and retention of skills.

## Data Availability Statement

The raw data supporting the conclusions of this article will be made available by the authors, without undue reservation.

## Ethics Statement

The studies involving human participants were reviewed and approved by University of Edinburgh and the University of Malawi—College of Medicine Research and Ethics Committee (COMREC) P08/19/2768. The patients/participants provided their written informed consent to participate in this study.

## Diplomatic Collaboration

Catherine Bamuya, Esmie Banda, James Boardman, Effie Chipeta, Mia Crampin, Sarah Cunningham Burley, Jean Desire Kabamba, Elizabeth Grant, Caroline Hollins Martin, Aisha Holloway, Khondwhani Kawaza, Corrine Love, Monica Malata, Fadhila Mazanderani, Catherine Mkandawire, Patrica Munthali, Peter Mwaba, Shakira Namisengo, Everist Njelesani, Linda Nyondo-Mipando, Hilary Pinnock, Muriel Syacumpi, Frank Taulo.

## Author Contributions

AV prepared the protocol for this study, with input and guidance from all authors. The education package was developed by AV, GM-G, DK, MM, BM, and LG, with GM-G, MM, EM, and LG co-ordinating the delivery of the training in Malawi. Data was analyzed by AV, who also prepared the first draft of the manuscript, under the guidance of SW, SJS, JN, DL, BM, BF, and LG. All authors provided critical insight for the manuscript.

## Funding

This research was funded by the National Institute for Health Research (NIHR) (GHR Project: 17/63/08 DIPLOMATIC collaboration) using UK aid from the UK Government to support global health research. We acknowledge the support of the Medical Research Council Centre for Reproductive Health (MRC CRH) Grant MR/N022556/1 and the support of the British Heart Foundation (RE/18/5/34216). SJS was supported by Wellcome Trust Clinical Career Development Fellowship 209560/Z/17/Z.

## Author Disclaimer

The views expressed in this publication are those of the authors and not necessarily those of the NIHR or the Department of Health and Social Care.

## Conflict of Interest

The authors declare that the research was conducted in the absence of any commercial or financial relationships that could be construed as a potential conflict of interest.

## Publisher's Note

All claims expressed in this article are solely those of the authors and do not necessarily represent those of their affiliated organizations, or those of the publisher, the editors and the reviewers. Any product that may be evaluated in this article, or claim that may be made by its manufacturer, is not guaranteed or endorsed by the publisher.

## Abbreviations

AC: Abdominal circumference
EDD: Estimated date of delivery
FL: Femur length
HC: Head circumference
ICC: Intraclass correlation coefficients
LMICs: Low- and middle- income countries
LMP: Last menstrual period
OSCE: Observed Structured Clinical Examinations
WHO: World Health Organization.
